# Involvement of cannabinoid receptors in depression of the putative nociceptive response in spinal cord preparations isolated from neonatal rats

**DOI:** 10.1186/s12576-023-00881-5

**Published:** 2023-10-06

**Authors:** Kayo Tsuzawa, Hiroshi Onimaru, Katsunori Inagaki, Masahiko Izumizaki

**Affiliations:** 1 Department of Orthopedic Surgery, Showa University School of Medicine, 1-5-8 Hatanodai, Shinagawa-Ku, Tokyo, 142-8555 Japan; 2 Department of Physiology, Showa University School of Medicine, 1-5-8 Hatanodai, Shinagawa-Ku, Tokyo, 142-8555 Japan

**Keywords:** Acetaminophen, Cannabinoid receptor, Lumbar spinal cord, Newborn rat

## Abstract

A metabolite of acetaminophen, AM404, which is an anandamide transporter inhibitor, induces analgesia mainly via activation of transient receptor potential channel 1 in the spinal cord, although the role of cannabinoid receptors remains to be studied. The ventral root reflex response induced by stimulation of the dorsal root in in vitro preparations of rat spinal cord is useful to assess the effect of analgesics. We analyzed the effects of AM404 and cannabinoid receptor antagonist AM251 on reflex responses in lumbar spinal cord preparations from newborn rats and found that the amplitude of the slow ventral root potential after administration of 10 µM AM404 was not significantly changed, whereas 10 µM AM251 significantly increased the amplitude. Administration of the cannabinoid receptor 1 agonist WIN55,212-2 (10 µM) did not significantly affect the reflex response. We suggest that endogenous cannabinoids in the spinal cord are involved in the antinociceptive mechanism through suppressive effects.

## Background

Nonsteroidal anti-inflammatory drugs (NSAIDs) and local anesthetics are mainly used for postoperative pain control in orthopedic surgery. In particular, multimodal analgesia using acetaminophen is a topic that has received attention in pain control because of the intravenous application of high-dose acetaminophen [1–3]. Some reports show that multimodal analgesia relieves postoperative pain and reduces the quantity of other drugs, such as NSAIDs (which are associated with the risk of peptic ulcer formation and renal dysfunction) or opioids (which are associated with adverse effects such as digestive symptoms, delirium, and respiratory depression) [4–6].

Regarding the analgesic mechanism of acetaminophen, the metabolite of acetaminophen, AM404 (N-arachidonoylphenolamine), which is known as an anandamide (arachidonoyl ethanolamide [AEA]) transporter inhibitor, is suggested to influence analgesic activity through several pathways, including transient receptor potential V1 (TRPV1), cyclooxygenase, cannabinoid receptor 1 (CB1), and serotonin neurons [6–10]. In the analgesic mechanism of acetaminophen in the higher brain, AM404 may promote activation of the central endocannabinoid system through action at both the TRPV1 channels and CB1 cannabinoid receptors [6]. Barrière et al. [11] reported that periaqueductal gray-located CB1 receptors were essential to exert the analgesic effects of acetaminophen through the AM404-activated TRPV1 channel-mGlu5 receptor-PLC-DAGL-CB1 receptor signaling cascade. At the spinal cord level, it was reported that AM404 caused analgesia via activation of TRPV1, and involvement of CB1 seemed to be less important (e.g., [10]). In the latter study, activation of TRPV1 by AM404 in slice experiments was produced within a range of minutes and was independent of CB1 receptors. Contrastingly, analgesia by systemic administration of acetaminophen appeared within a range of hours, and it was not clear whether the action of AM404 as an anandamide transporter inhibitor was involved in the long-lasting effects.

As an experimental model for neurophysiological and neuropharmacological studies of the pain control mechanism, Otsuguro et al. [12] reported that the nociceptive response could be evaluated by the slow ventral root potential (sVRP) induced by stimulation of the dorsal root in in vitro preparations of rat lumbar spinal cord. These methods enable stable recording of the nociceptive response over a longer period (hours) [13, 14]. Thus, this in vitro experimental model is useful for assessing the analgesic effects of drugs. The objective of the present study was to clarify the effects of AM404 and the cannabinoid receptor antagonist AM251 on the sVRP and to verify the relevance of the CB1 receptor to the analgesic mechanism.

## Methods

The experimental protocols were approved by the Animal Research Committee of Showa University (approval nos. 09049, 02022, 03066) in accordance with Law No. 105 for the care and use of laboratory animals of the Japanese government. All efforts were made to minimize the number of animals used and their suffering.

### Preparation and solutions

Spinal cord preparations (the tenth thoracic–fifth lumbar spinal cord) were dissected from 0- to 3-day-old Wistar rats (n = 27, either sex) deeply anesthetized with isoflurane as previously described [13–15]. Preparations were continuously superfused with the following artificial cerebrospinal fluid (ACSF) [16] (mM): 124 NaCl, 5.0 KCl, 1.24 KH_2_PO_4_, 2.4 CaCl_2_, 1.3 MgCl_2_, 26 NaHCO_3_, and 30 glucose equilibrated with 95% O_2_ and 5% CO_2_, pH 7.4, at 25–27 °C. The antinociceptive effects of the examined drugs were evaluated by recording the fourth or fifth lumbar spinal cord reflex following ipsilateral, same-level dorsal root stimulation via a glass suction electrode. The reflex response was recorded via the 0.5 Hz high-pass filter of an AC amplifier (AB-651 J, Nihon Kohden, Tokyo, Japan). Stimulation (5–20 V, 200 μs square pulse, every 60 s) was applied to the dorsal root with an intensity that was set to produce an approximate half-maximum response. The preparations were superfused with ACSF for at least 15 min until the spinal reflex stabilized.

### Drugs

AM404 and AM251 (CB1 antagonists) were purchased from Sigma-Aldrich (Tokyo, Japan), and WIN55,212-2 (CB1 agonist) was purchased from ChemScene (Monmouth Junction, NJ, USA). Drugs were stored as a 10 mM stock solution. Effective concentrations of AM404 in the in vitro experiments have been reported to be 3–30 μM, e.g., in lumbar dorsal root ganglion neurons from adult mice [17] and slice preparations from adult rat lumbar cords [10]. In our preliminary experiments, therefore, we examined the effects of 10 and 30 μM AM404 on the sVRP. Because we found that there was no difference in the response between the two concentrations, data in the present study were collected using only 10 μM AM404. Previous studies reported that 10 μM WIN55,212-2 and 10 μM AM251 were effective in depressing and enhancing spinal seizure-like activity, respectively, in the brainstem-spinal cord preparation from newborn rat [18, 19]. Thus, all drugs were dissolved in ACSF and bath-applied at a final concentration of 10 μM.

### Data analysis

All data analyses were performed using the LabChart 7 Pro software program (ADInstruments, Castle Hill, Australia). The peak amplitude of the sVRP was calculated from the mean value of five consecutive responses in each preparation. Data are presented as the mean and standard deviation (SD). The significance of the values was analyzed by a one-way repeated-measures analysis of variance (ANOVA) followed by a Tukey–Kramer multiple comparisons test (GraphPad InStat; GraphPad Software Inc., La Jolla, CA, USA). *P* values of < 0.05 were considered to indicate statistical significance.

## Results

### Effects of AM404 on the sVRP

**Fig. 1:**
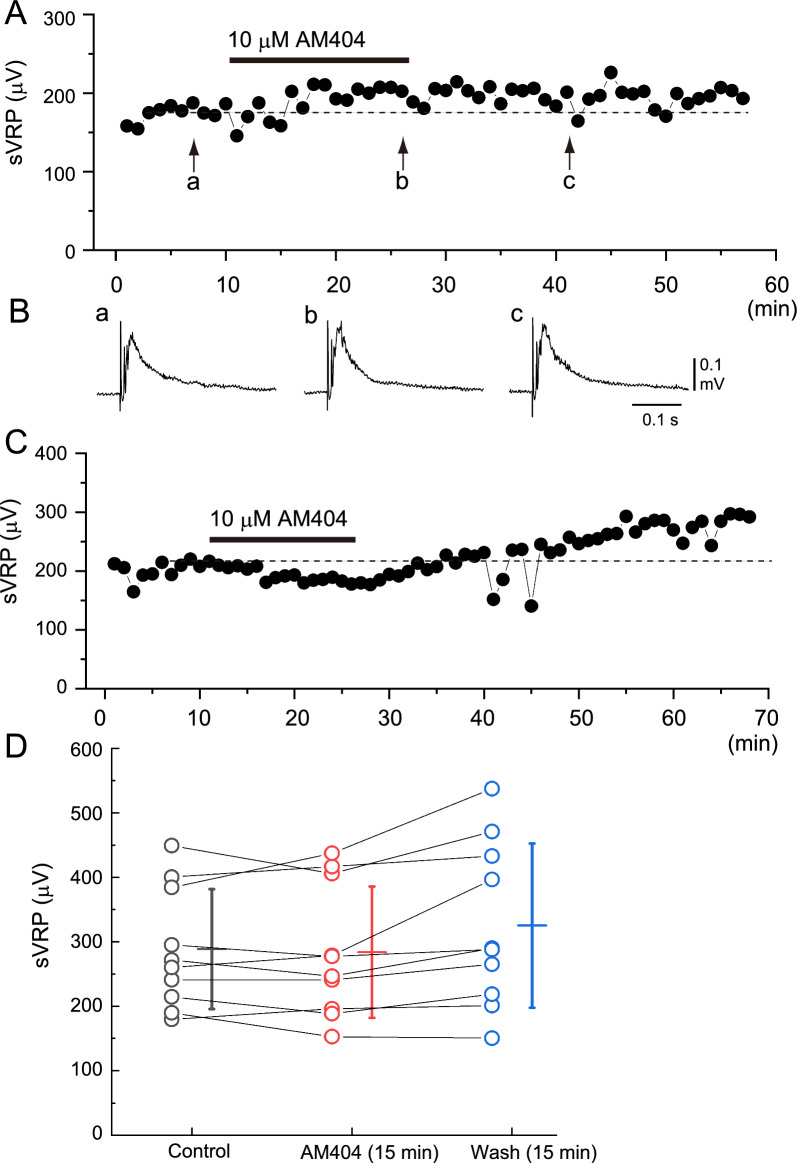
Typical example of the effect of AM404 on the slow ventral root potential (sVRP) in newborn rat spinal cord preparations. **A** Time course of the changes in peak amplitude of the sVRP in response to the application of 10 µM AM404. **B** Examples of the reflex response in the control (a), at 15 min after the administration of AM404 (b), and after 15 min of washout (c). a–c correspond to a-c in panel **A**. **C** Example showing a decrease in sVRP amplitude during the application of AM404 and an increase after washout. **D** Data plots of the sVRP amplitude from 10 preparations at control (black), 15 min application of AM404 (red), and 15 min after washout (blue). Bars on the right side of the individual datasets denote the mean ± S.D. The average values of the sVRP amplitude did not change significantly

The effects of a 15 min application of 10 µM AM404 on the spinal reflex response were examined in 10 preparations. The average peak amplitudes of the sVRP were 288.5 ± 93.1 µV, 283.9 ± 101.8 µV, and 325.1 ± 127.6 µV at control, immediately before washout, and 15 min after washout, respectively. A typical example and summary are shown in Fig. 1. Although the changes in the average sVRP amplitude in the 10 preparations were not statistically significant, in half of the preparations, they decreased during the application of AM404 and tended to increase after washout. Although our results did not show that the increase after washout always occurred in association with the decrease during AM404 application (Fig. 1D), Fig. 1C shows an example in which both changes (i.e., decrease in amplitude → increase in amplitude) were observed.

### Effects of AM251 on the sVRP

**Fig. 2:**
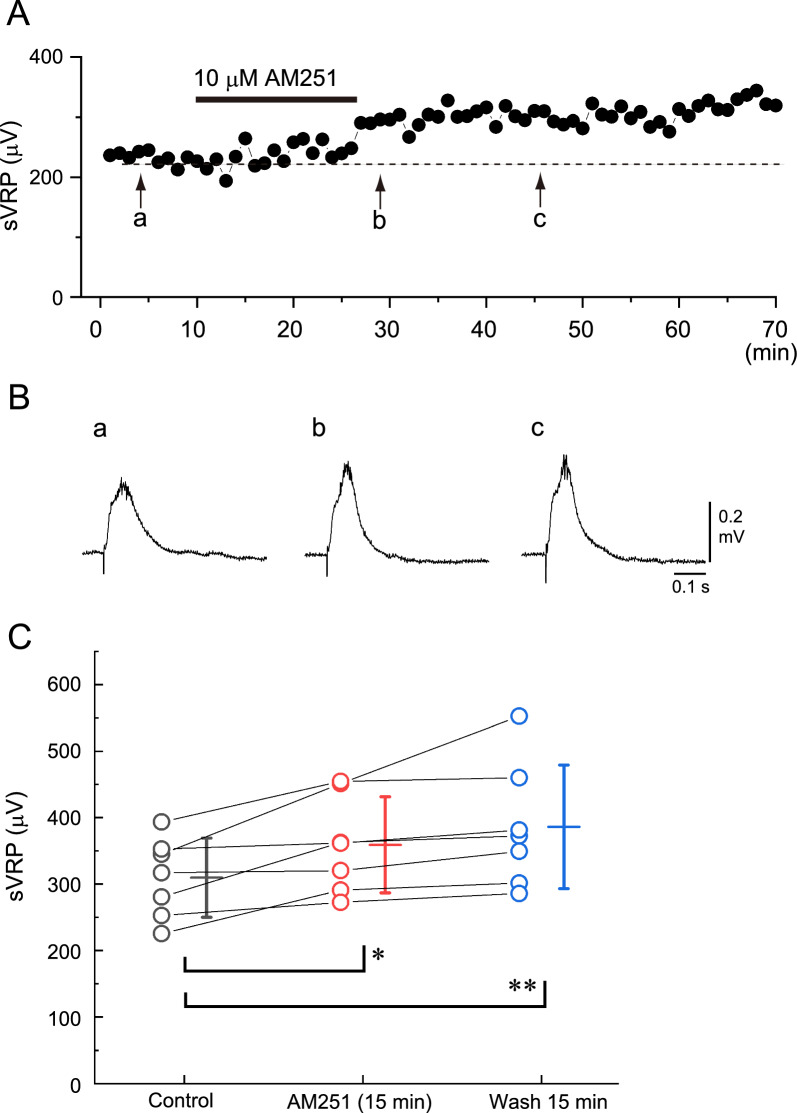
Typical example of the effect of AM251 on the slow ventral root potential (sVRP) in newborn rat spinal cord preparations. **A** Time course of changes in the peak amplitude of the sVRP in response to the application of 10 µM AM251. **B** Examples of the reflex response in control (a), at washout immediately after the 15 min administration of AM251 (b), and after 15 min washout (c). a–c correspond to a-c in panel **A**. Note the gradual increase in the sVRP amplitude after treatment with AM251. **C** Data plots of the sVRP amplitude from 7 preparations at control (black), 15 min application of AM404 (red), and 15 min after washout (blue). Bars on the right side of the individual datasets denote the mean ± S.D. The average values of the sVRP amplitude were significantly increased. ** *P* < 0.01, *** *P* < 0.001, by a one-way repeated-measures ANOVA, followed by a Tukey‒Kramer multiple comparisons test

The effects of the CB1 antagonist 10 µM AM251 on the spinal reflex response were examined in 7 preparations. The peak amplitude of the sVRP gradually increased after the administration of AM251, and this tendency lasted for more than 30 min after washout. The average peak amplitudes of the sVRP were 309.6 ± 59.6 µV, 359.1 ± 72.2 µV, and 386.1 ± 93.0 µV at control, immediately before washout (*P* < 0.05, in comparison to control), and 15 min after washout (*P* < 0.01, in comparison to control), respectively. A typical example and summary are shown in Fig. 2.

### Effects of the CB1 agonist WIN55,212–2 on the sVRP

**Fig. 3:**
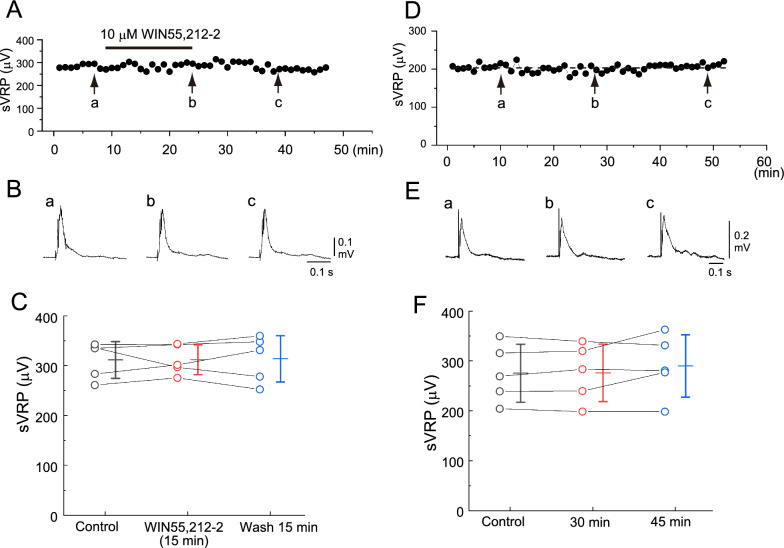
Typical example of the effect of WIN55,212-2 and time control on the slow ventral root potential (sVRP) in newborn rat spinal cord preparations. **A** Time course of changes in the peak amplitude of the sVRP in response to the application of 10 µM WIN55,212-2. **B** Examples of the reflex response in control (a), at 15 min after the administration of WIN55,212-2 (b), and after 15 min of washout (c). a–c correspond to a-c in panel **A**. **C** Data plots of the sVRP amplitude from 5 preparations at control (black), 15 min application of WIN55,212-2 (red), and 15 min after washout (blue). Bars on the right side of the individual datasets denote the mean ± S.D. The average values of the sVRP amplitude did not change significantly. **D** Time course of the changes in the peak amplitude of sVRP without drug application. **E** Examples of the reflex response. a-c correspond to a-c in panel **A**. **F** Data plots of the sVRP amplitude from 5 preparations at control (black), 30 min after control data acquisition (red), and 45 min after control data acquisition (blue). Bars on the right side of the individual datasets denote the mean ± S.D. The average values of the sVRP amplitude did not change significantly

Next, we tested the effects of the CB1 agonist WIN55,212-2 on the spinal reflex response in 5 preparations. The administration of 10 µM WIN55,212-2 showed no significant effect on the reflex response. The average peak amplitudes of the sVRP were 311.5 ± 36.9 µV, 311.9 ± 29.9 µV, and 313.8 ± 46.4 µV at control, immediately before washout, and 15 min after washout, respectively. A typical example and summary are shown in Fig. 3A–C.

### Time control

For time control, we examined the peak amplitude of the sVRP in 5 preparations under ACSF without the administration of any drugs. There was no significant difference in the peak amplitude for more than 45 min. The average peak amplitudes of the sVRP were 275.4 ± 58.2 µV, 276.1 ± 57.7 µV, and 290.0 ± 62.6 µV at 15, 30, and 45 min, respectively, after the measurement was started. A typical example and summary are shown in Fig. 3D–F.

## Discussion

In the present study, we investigated the effects of an anandamide transporter inhibitor, AM404, and a CB1 receptor antagonist, AM251, on the sVRP in newborn rat spinal cord preparations. Our results showed that AM404 exerted no significant effect, whereas AM251 induced a long-lasting increase in the amplitude of the sVRP.

Acetaminophen is metabolized into the N-acylphenolamine AM404 in the liver, brain, or spinal cord [6]. Zygmunt et al. [20] reported that AM404 affected the activation of vanilloid receptors (TRPV1). Ohashi et al. [10] suggested that AM404 induces analgesia directly via TRPV1 expressed on the central terminals of C-fibers in the spinal dorsal horn but not via CB1. They showed that AM404 decreased the amplitudes of excitatory post-synaptic currents (EPSCs) evoked by C-fiber stimulation. This AM404-induced response was blocked in the presence of the TRPV1 receptor antagonist capsazepine (10 μM) and was preserved in the presence of the CB1 receptor antagonist AM251 (3 μM) [10, 21]. However, it might be not clear how a transient activation of TRPV1 by AM404 relates to the long-lasting analgesic effects induced by systemic administration of acetaminophen in vivo as shown in Ohashi et al. [10] because these responses showed different time courses, and TRPV1 might be desensitized rapidly [22]. Sustained desensitization of TRPV1 following initial activation by AM404 might be related to the long-lasting analgesic effects provided by acetaminophen [10]. We previously showed that the application of a TRPV1 agonist, capsaicin, induced a transient decrease in the amplitude of the sVRP followed by recovery, even during capsaicin treatment, that was possibly due to the desensitization of TRPV1 [15, 23]. Therefore, if AM404 could act via TRPV1 in the present spinal cord preparation, it would be expected to decrease the amplitude of the sVRP. Indeed, we observed a decrease in the sVRP amplitude in half of the preparations during AM404 application, although these changes in the average value of all 10 preparations did not reach statistical significance. One possible explanation for our result—that AM404 did not effectively activate TRPV1—might be differences in the ages of the rats that were used (i.e., newborn rats in the present study vs. adult rats by Ohashi et al. [10]. In contrast, the administration of AM251 increased the amplitude of the sVRP. This result could be explained by the mechanism through which endogenous anandamide, AEA, was already functioning suppressively under control conditions, and the blockade of CB1 receptors by AM251 reduced the suppressive effect of endogenous AEA. Thus, the putative increase in AEA by the inhibitory effect of AM404 on the anandamide transporter showed no further remarkable effect. A similar mechanism could also explain why the CB1 agonist WIN55,212-2 induced no significant effect. We speculated that partial activation of the CB1 receptor by endogenous anandamide is enough to cause reduction of the sVRP, and this effect may be saturated under the present experimental condition. Therefore, further activation of CB1 by WIN55,212-2 might not induce any detectable changes in the sVRP. Thus, our results suggest that endogenous cannabinoids suppress the nociceptive reaction in the spinal cord. Future studies will need to clarify whether the endocannabinoid system in the spinal cord is involved in analgesia induced by AM404 (and thus by acetaminophen) under certain conditions.

In the present study, we observed a slow increase in amplitude after the washout of AM404 in some preparations. Although the results suggest the presence of slowly appearing effects of AM404 in some preparations, the detailed mechanism is unknown.

An sVRP is induced by the excitation of the ventral root motoneuron via plural interneurons. On the spinal dorsal horn, glutamic acid or substance P released from the primary afferent Aδ and C fibers affects these interneurons [24], and stimulation of the CB1 receptors may suppress glutamatergic EPSCs in the substantia gelatinosa neurons of the rat spinal cord [25, 26]. Therefore, the target sites of AM251 were thought to be primarily located in the dorsal horns of the spinal cord. It has been suggested that AM404 activates CB1 receptors and/or TRPV1 channels in the periaqueductal gray of the midbrain and induces analgesic effects through descending serotonergic pathways [6]. However, this system could not be involved in the present experiments, which used preparations of the 10th thoracic–5th lumbar spinal cord. We recently reported that CB1 receptors may be involved in the descending inhibitory system at the motor neuron level against the generation of spinal seizure-like activity [19]. Such putative CB1 receptors in the ventral horn could be an additional target of AM215.

## Conclusions

We found that the sole administration of AM404 was not associated with a remarkable change in the sVRP in newborn rat spinal cord preparations. However, the administration of AM251 induced long-lasting increasing effects on the amplitude of the sVRP. The findings of the present study suggest that CB1 receptors could be involved in the intrinsic antinociceptive mechanism.

## Data Availability

The datasets used in the present study are available from the corresponding author upon reasonable request.

## Abbreviations

ACSF: Artificial cerebrospinal fluid
AEA: Arachidonoyl ethanolamide
AM404: N-arachidonoylphenolamine
ANOVA: Analysis of variance
CB1: Cannabinoid receptor 1
EPSCs: Excitatory post-synaptic currents
NSAIDs: Nonsteroidal anti-inflammatory drugs
sVRP: Slow ventral root potential
TRPV1: Transient receptor potential V1
